# Efficacy of granulocyte colony-stimulating factor for infertility undergoing IVF: a systematic review and meta-analysis

**DOI:** 10.1186/s12958-023-01063-z

**Published:** 2023-04-03

**Authors:** Lu-lu Fu, Ying Xu, Jing Yan, Xue-ying Zhang, Dan-dan Li, Lian-wen Zheng

**Affiliations:** Reproductive Medical Center, The Second Hospital of Jilin University, Changchun, China

**Keywords:** Granulocyte colony-stimulating factor, IVF, Thin endometrium, Repeated implantation failure, Recurrent spontaneous abortion

## Abstract

**Objective:**

This study aimed to evaluate the effectiveness of granulocyte colony-stimulating factor (G-CSF) for infertility and recurrent spontaneous abortion.

**Methods:**

Existing research was searched in PubMed, Embase and Cochrane Library till Dec 2021. Randomized control trials (RCTs) that compared G-CSF administration with the control group in infertility women undergoing IVF were included. The primary outcomes included clinical pregnancy rate; the secondary outcomes included live birth rate, abortion ratebiochemical pregnancy rate, embryo implantation rate, as well as endometrial thickness.

**Result(s):**

20 RCTs were included in this study. G-CSF increased the clinical pregnancy rate (RR = 1.85; 95% CI: 1.07, 3.18) and the endometrial thickness (MD = 2.25; 95% CI: 1.58,2.92;) in patients with thin endometrium undergoing IVF. G-CSF increased the biochemical pregnancy rate (RR = 2.12; 95% CI: 1.54, 2.93), the embryo implantation rate (RR = 2.51; 95% CI: 1.82, 3.47) and the clinical pregnancy rate (RR = 1.93; 95% CI: 1.63, 2.29) in patients with a history of repeated implantation failure undergoing IVF. No differences were found in pregnancy outcomes of general IVF patients.

**Conclusions:**

Granulocyte colony-stimulating factor is likely to be a potential option for infertility women undergoing IVF with thin endometrium or recurrent implantation failure .

**Trial registration:**

Retrospectively registered (The PROSPERO registration number: CRD42022360161).

**Supplementary Information:**

The online version contains supplementary material available at 10.1186/s12958-023-01063-z.

## Introduction

Infertility has an effect on approximately 10–15% of couples worldwide. Over the past few decades, pregnancy rates have increased significantly due to the advances in assisted reproduction techniques (ART). However, it is still difficult for numerous couples to obtain a live birth, especially for infertility women with thin endometrium, repeated implantation failure and recurrent spontaneous abortion, which remains a huge challenge for clinicians. Under the mentioned context, immunotherapy may be considered one of the potentially effective treatments to improve uterine receptivity, facilitate implantation and prevent abortion in the above women [1] .

G-CSF refers to a glycoprotein synthesized by bone marrow cells, stromal cells, mononuclear cells, fibroblasts, natural killer (NK) cells and endometrial cells. G-CSF is primarily capable of stimulating the proliferation and differentiation of neutrophils in the bone marrow and controlling their release to the bloodstream [2]. In 1983, G-CSF was first recognized and purified in mice, and the human form of G-CSF was cloned three years later in 1986 [3, 4]. Originally, recombinant human G-CSF has been largely adopted to treat haematological disorders [5, 6]. Over the past few years, G-CSF quantification in follicular fluid has been confirmed as a useful biomarker of oocyte competence. Ledee et al. [7] examined the level of G-CSF in follicular fluid from 78 patients undergoing IVF. Embryos derived from higher G-CSF follicles tend to have a higher implantation rate than those with lower G-CSF follicles, suggesting that G-CSF may be involved in reproduction. Furthermore, over the past few years, numerous publications, including several systematic reviews and meta-analyses, have revealed that G-CSF plays an effective role in pregnancy success [8–29].

Eftekhar M et al. [16] concluded in a review that G-CSF played a certain role in ovulation, luteinized unruptured follicle syndrome, and improved poor ovarian responders and endometrial receptivity. Kamath MS et al. [8] recently evaluated the use of G-CSF in IVF cycles in a meta-analysis published in 2020 (including 12 RCTs, the G-CSF group = 522, as well as the control group = 528). They suggested that G-CSF administration might increase the clinical pregnancy rate in women with a history of RIF (RR = 2.10, 95% CI: 1.53, 2.89). For all IVF women or those with thin endometrium, whether the administration of G-CSF increases the ongoing pregnancy rate or the overall clinical pregnancy rates, or decreases the abortion rate compared with the control group remains uncertain. The above studies have drawn inconsistent conclusions and produced limited scientific evidence, whether G-CSF has a positive effect on all women with fertility problems remains unclear. Accordingly, this study aimed to evaluate the effect of G-CSF on women undergoing IVF with thin endometrium, repeated implantation failure, recurrent spontaneous abortion or not.

## Methods

### Literature search methodology

This meta-analysis was conducted in accordance with the Preferred Report Item for Systematic Reviews and Meta-analyses statement [30]. Randomized controlled trials (RCTs) were searched on PubMed, EMbase and Cochrane Library until Dec 2021. Key words used for search included “G-CSF”, “granulocyte colony-stimulating factor”, “IVF”, “in vitro fertilization”, or “ICSI”, “intracytoplasmic sperm injection”, or “RIF”; “repeated implantation failure”, or “thin endometrium”, or “unresponsive endometrium” or “RSA”, “recurrent spontaneous abortion”, or “RPL”, “recurrent pregnancy loss”. Moreover, the original text was also searched by references in the literatures to avoid missing suitable studies. This search was conducted independently by Fu LL and Li DD.

### Study selection

This study followed the study protocol for the review in terms of PICOS. Studies were included if the target population were women undergoing IVF with thin endometrium, repeated implantation failure, recurrent spontaneous abortion or not who were given G-CSF in the intervention group and placebo, or no treatment was given in the control group. The subgroup analysis was divided into four groups, IVF without special selection named general IVF, IVF with a history of thin endometrium named IVF with thin endometrium, IVF with a history of repeated implantation failure named IVF with RIF, and IVF with a history of recurrent spontaneous abortion named IVF with RSA. The primary outcome measure was clinical pregnancy rate (CPR). The secondary outcome measure included live birth rate (LR) or ongoing pregnancy rate (OPR), abortion rate (AR), biochemical pregnancy rate (BPR), embryo implantation rate (ER) and endometrium thickness. Only RCTs were included in this study, and a meta-analysis was performed if appropriate. Case reports and non-randomized studies including case-control studies and cohort studies were excluded since they are related to a higher risk of bias. The above work was conducted independently by Fu LL and Li DD. Any disagreements relating to inclusion would be resolved through consensus with a third reviewer (Xu Y).

### Evaluation of methodological quality and data extraction

The selected studies were independently evaluated by two authors (Fu LL and Li DD) for methodological quality and data extraction. The methodological quality for risk of bias was evaluated based on the Cochrane risk of a bias evaluation tool (www.training.cochrane.org/handbook). The evaluation included random sequence generation, allocation concealment, blinding of participants and personnel, blinding of outcome evaluators, incomplete outcome data, selective reporting, as well as other biases, which are presented in the risk of bias graphs and summaries. Two review authors (Fu LL and Li DD) independently extracted detailed data from eligible studies. Any disagreements were resolved by a third author (Xu Y).

### Statistical analysis

For dichotomous data, the numbers of events in the G-CSF group and the control groups of the respective study were adopted to obtain the Mantel-Haenszel risk ratio (RR). For quantitative results, it was expressed as the mean ± SD. *P* value was evaluated statistically with the I^2^ statistic. If the I^2^ value was > 50%, with higher heterogeneity, a random effects model would be applied. If the I^2^ value was < 50%, with lower heterogeneity, a fixed effects model would be used. 95% confidence intervals (CIs) were presented for all outcomes. Potential publication bias was examined qualitatively using the funnel plot. Statistical analyses were conducted with RevMan 5.3 software (Cochrane Collaboration, Oxford, UK).

## Result

### Study characteristics

**Fig. 1:**
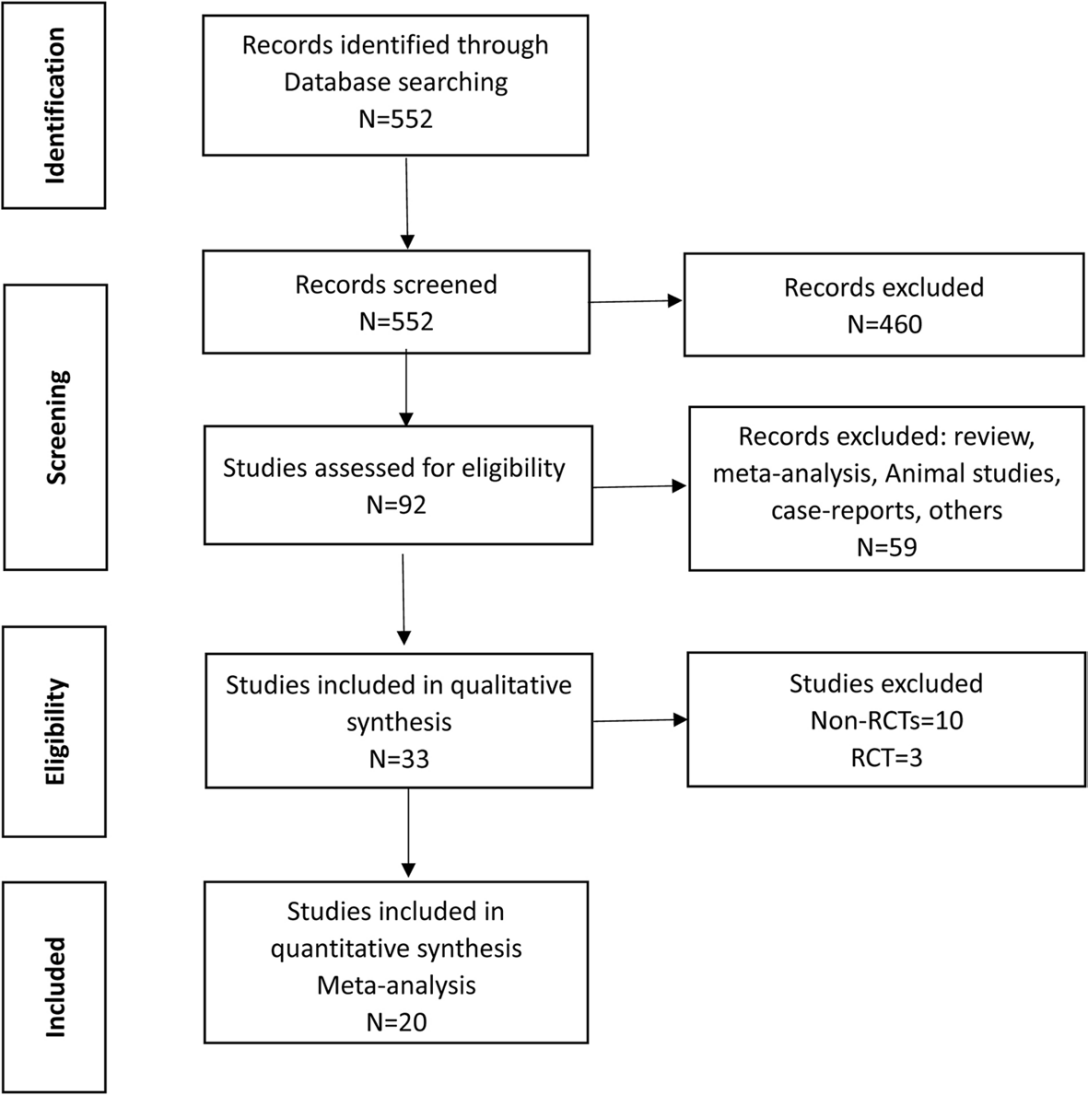
PRISMA flow diagram of study selection

A total of 20 RCTs screened from 552 studies were included in this meta-analysis (3 general IVF, 3 IVF with thin endometrium, 14 IVF with RIF and 0 IVF with RSA). The search results are summarized in Fig. 1. The included studies above totally enrolled 1966 participants (999 women were randomly allocated to treatment with G-CSF and 967 women were randomly allocated to placebo or no treatment.). A total of 13 of the above trials were published as full articles [31–43], seven were conference abstracts [44–50]. Eight trials were conducted in the Iran, three in India, three in Italy, one in Germany, one in Turkey, one in Russia，one in China, one in Egypt and one in USA.The sample size per study ranged from 28 to 157 participants. The detailed characteristics of the included studies above are shown in Supplemental Table 1. A total of 13 studies [51–63] with the effect of G-CSF on pregnancy outcomes in women were excluded in the study (Supplemental Table 3). The summarized effectiveness of G-CSF on pregnancy outcomes is listed in Supplemental Table 2**.**

### Risk of bias in included studies

Judgments about each risk of bias item presented as percentages across all included RCTs are shown in Supplemental Fig. 1. Each risk of bias item for each RCT are presented in Supplemental Fig. 2.

### Clinical pregnancy rate

**Fig. 2:**
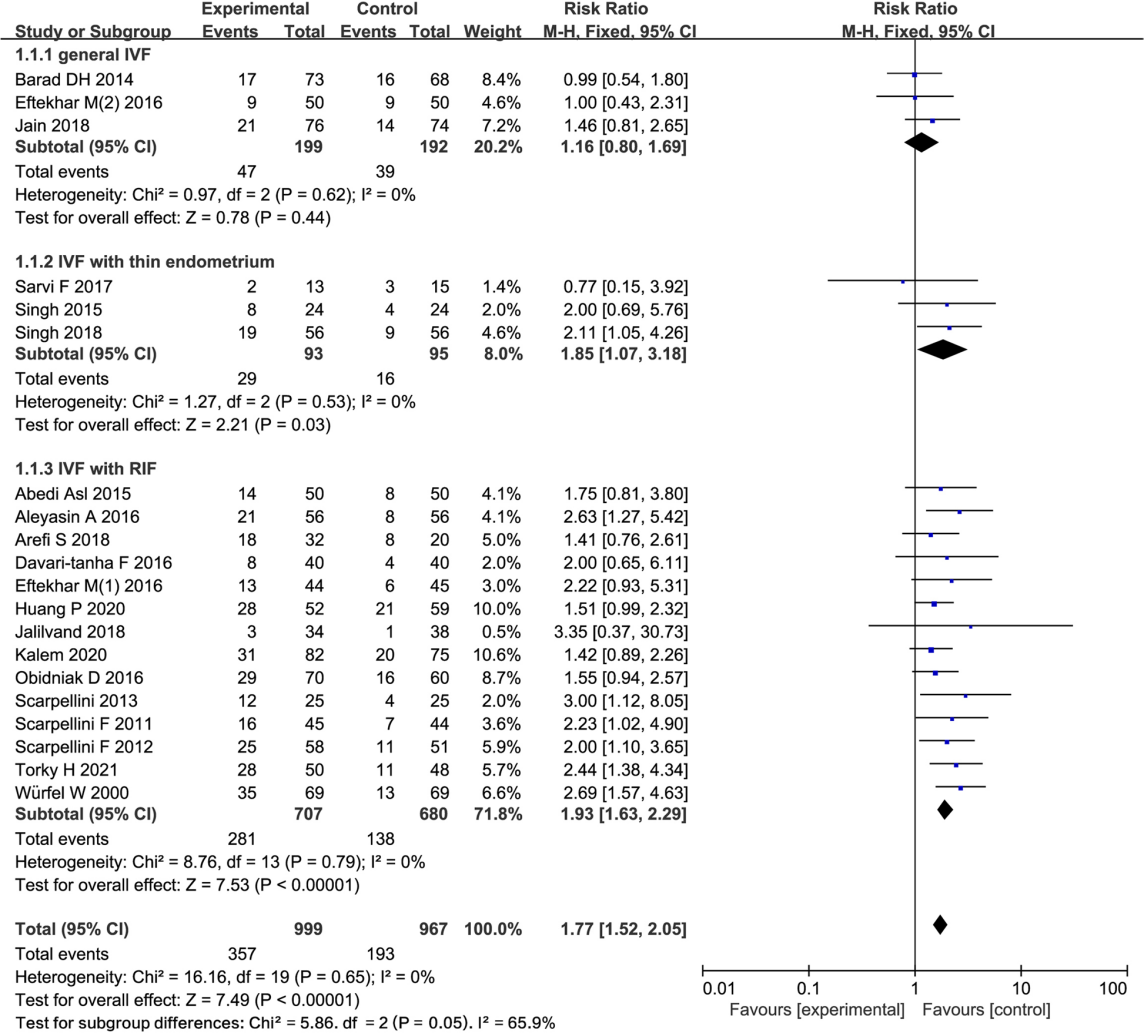
Forest plot of comparison: G-CSF vs control, outcome: Clinical pregnancy rate

20 studies reported CPR in 1966 women (3 general IVF, 3 IVF with thin endometrium, 14 IVF with RIF). The pooling results indicated that, CPR was higher in the G-CSF group compared with the control group(RR = 1.77; 95% CI: 1.52, 2.05; Fig. 2). The subgroup analysis suggested that three studies reported CPR in 391 women in general IVF subgroup, and no difference was found between the above two groups (RR = 1.16; 95% CI: 0.8, 1.69 Fig. 2). Three studies reported CPR in 188 women in IVF with thin endometrium subgroup, CPR was higher in the G-CSF group compared with the control group(RR =1.85; 95% CI: 1.07, 3.18; Fig. 2**)**. 14 studies reported CPR in 1387 women in IVF with RIF subgroup, CPR was higher in the G-CSF group compared with the control group(RR = 1.93; 95% CI: 1.63, 2.29; Fig. 2). In women undergoing IVF with RIF, a subgroup analysis was further conducted based on the route of G-CSF administration (intrauterine injection *n* = 7, subcutaneous injection *n* = 8). There was an increased CPR through both intrauterine injection (RR = 1.71; 95% CI: 1.35, 2.16) and subcutaneous injection (RR = 2.13; 95% CI: 1.68, 2.69; Supplemental Fig. 3); Moreover, a subgroup analysis was conducted in accordance with the embryo transfer cycle of ET or FET (ET n = 7, FET *n* = 3, unknown *n* = 4). There was an increased CPR in all transfer cycle (ET: RR = 1.98; 95% CI: 1.55, 2.54; FET: RR = 1.58; 95% CI: 1.18, 2.11; unknown: RR = 2.19; 95% CI: 1.56, 3.08; Supplemental Fig. 4).

### Live birth rate

**Fig. 3:**
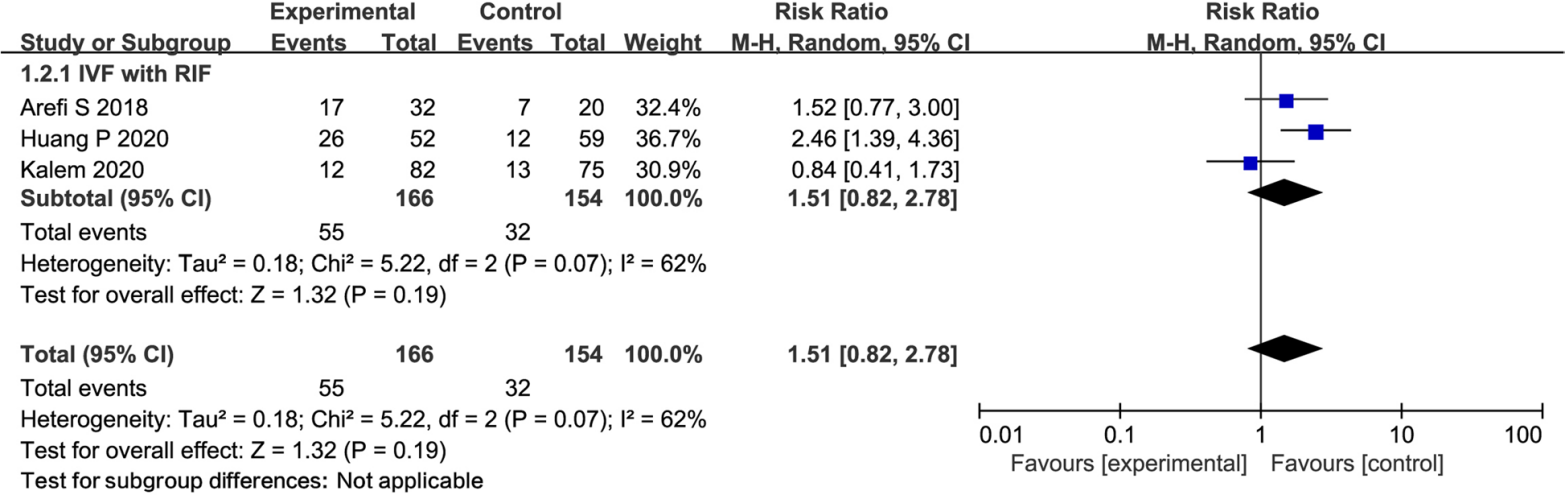
Forest plot of comparison: G-CSF vs control, outcome: Live birth rate

Three studies reported LR in 320 women undergoing IVF with a history of RIF. There was no difference between the G-CSF group and the control group (RR = 1.51; 95% CI: 0.82, 2.78; Fig. 3).

#### Abortion rate.

**Fig. 4:**
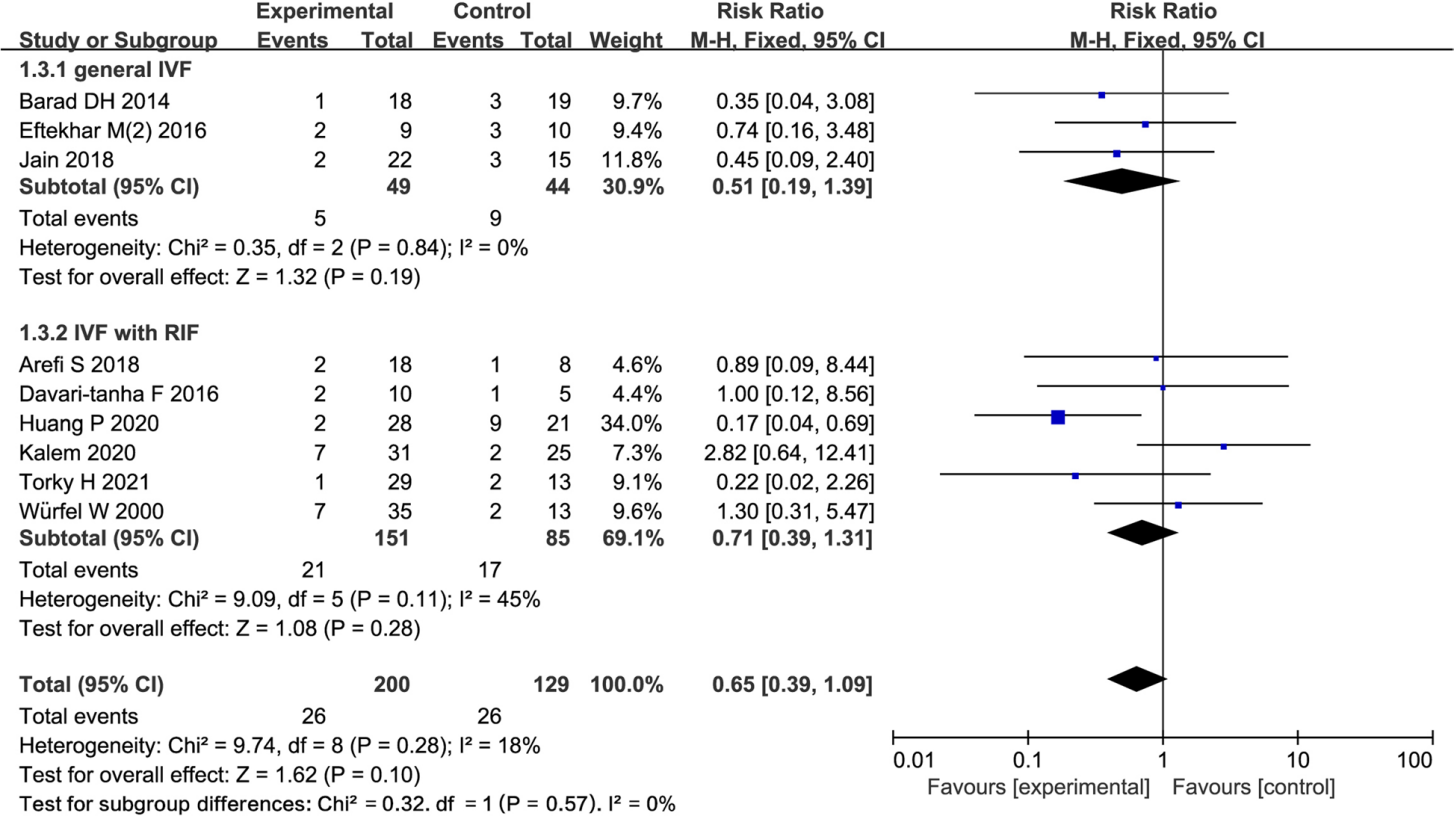
Forest plot of comparison: G-CSF vs control, outcome: Abortion rate

Nine studies reported AR in 377 women (3 general IVF, 6 IVF with RIF). The pooling results indicated that, there was no difference in the G-CSF group compared with the control group (RR = 0.39; 95% CI: 0.39, 1.09; Fig. 4). The subgroup analysis suggested that three studies reported AR in 90 women in general IVF subgroup, and no difference was found between the G-CSF group and the control group (RR = 0.51; 95% CI: 0.19, 1.39; Fig. 4). Six studies reported AR in 287 women in IVF with RIF subgroup, no difference was found between the two groups (RR = 0.71; 95% CI:0.39, 1.31; Fig. 4).

### Ongoing pregnancy rate

Only two studies reported OPR in 250 women in general IVF group. No difference was found between the G-CSF group and the control group (RR = 1.4; 95% CI: 0.82, 2.37; Supplemental Fig. 5).

### Biochemical pregnancy rate

Seven studies reported BPR in 781 women (3 general IVF, 4 IVF with RIF). The pooling results revealed that BPR was higher in the G-CSF group compared with the control group (RR = 1.56; 95% CI: 1.24,1.98; Supplemental Fig. 6). The subgroup analysis suggested that three studies reported BR in 391 women in general IVF subgroup, and no difference was found between the above two groups (RR = 1.07; 95% CI: 0.75,1.53; Supplemental Fig. 6). Four studies reported BPR in 390 women in IVF withRIF subgroup, BPR was significantly higher in the G-CSF group (RR = 2.12; 95% CI: 1.54, 2.93; Supplemental Fig. 6). Embryo implantation rate.

Eight studies reported ER in 1879 women (2 general IVF, 1IVF with thin endometrium, 5 IVF with RIF). The pooling results indicated that, ER was higher in the G-CSF group compared with the control group(RR = 1.82; 95% CI: 1.22, 2.70; Supplemental Fig. 7). The subgroup analysis suggested that two studies reported ER in 688 women in general IVF subgroup, no difference was found between the above two groups (RR = 0.99; 95% CI: 0.57, 1.72; Supplemental Fig. 7). Only one study reported ER in 85 women in IVF with thin endometrium, there were no differences (RR = 1.93; 95% CI: 0.42, 8.97; Supplemental Fig. 7). Five studies reported ER in 1106 women in IVF with RIF, G-CSF significantly improved ER (RR = 2.51; 95% CI: 1.82, 3.47; Supplemental Fig. 7).

### Endometrium thickness

Ten studies enrolled 1034 participants measured endometrium thickness (3 general IVF, 2 IVF with thin endometrium, 5 IVF with RIF). The pooling results indicated that there wasno difference in endometrium thickness between the above two groups (MD = 0.28; 95% CI: − 0.23, 0.79; Supplemental Fig. 8). The subgroup analysis suggested that three studies reported endometrium thickness in 391 women in general IVF subgroup, no difference was found between the G-CSF group and the control group (MD = -0.08; 95% CI: − 0.44,0.27; Supplemental Fig. 8). Two studies reported endometrium thickness in 76 women in IVF with thin endometrium subgroup, endometrium thickness was significantly increased in the G-CSF group compared with the control group(MD = 2.25; 95% CI: 1.58, 2.92; Supplemental Fig. 8). Five studies reported endometrium thickness in 567 women in IVF with RIF subgroup, no difference was found between the above two groups (MD = -0.16; 95% CI: − 0.48, 0.15; Supplemental Fig. 8).

### Publication bias analysis

For publication bias, the funnel plots indicate a relatively low likelihood of publication bias as presented in Supplemental Fig. 9.

## Discussion

This study has been the most comprehensive meta-analysis about G-CSF and pregnancy outcomes thus far, which included general IVF patients, IVF with thin endometrium and IVF with repeated implantation failures. A total of 20 RCTs of relatively high quality were included, and it was concluded that G-CSF could have a positive effect on IVF patients with thin endometrium or repeated implantation failure.

### The use of G-CSF in women undergoing IVF

In this meta-analysis, three RCT studies included 199 patients in the G-CSF group and 192 patients in the control group undergoing IVF without special selection was defined as the general IVF. No benefit was found using G-CSF in general IVF patients in terms of CPR, AR, ER, BPR, OPR and endometrium thickness. Barad DH et al. [31] first reported that G-CSF did not affect endometrial thickness, ER and CPR in the above women enrolled in the study. Jain S et al. [33] claimed that no difference was found between pregnancy rate and endometrial thickness, whereas the endometrial vascularity significantly improved on the day of embryo transfer in the G-CSF group. Zhao J et al. [23] first claimed that G-CSF administrated subcutaneously increased the pregnancy rate (OR 3.12,95% CI 1.67,5.81) and ER (OR 2.82, 95% CI 1.29,6.15) compared with the control group in women undergoing IVF in a meta-analysis published in 2016. Studies by Zhao J et al. included women undergoing general IVF, women with thin endometrium and women with a history of RIF. The huge heterogeneity may lead to the inconsistency with this study. Furthermore, in 2009 Scarpellini F et al. [60] measured the effect of G-CSF in low-responder women undergoing IVF in one excluded RCT in this meta-analysis. The quality of oocytes and the ability to be fertilized was correlated with G-CSF intrafollicular level. G-CSF could be effective to improve the results in low responder women undergoing IVF. It may require more well-designed RCTs with larger sample sizes to explore the effect of G-CSF in general IVF women.

### G-CSF in women with thin endometrium undergoing IVF

Suitable thickness of endometrium takes on a critical significance to embryo implantation and pregnancy success [64]. When the endometrium was less than 7 mm or more than 14 mm, it was generally related to poor pregnancy outcomes [65–67]. In this meta-analysis, if endometrial thickness was < 7 mm in two studies, and < 6 mm in one study on the day of trigger was defined as thin endometrium. CPR was significantly higher in the G-CSF group compared with the control group. The endometrial thickness significantly increased in patients with thin endometrium undergoing IVF in the G-CSF group.

The first time G-CSF was adopted to improve endometrium thickness in women undergoing IVF with thin endometrium was explored by Gleicher et al. [68] in 2011. Four patients were treated with intrauterine infusions of G-CSF and all got pregnancy. Xie et al. [13] in a meta-analysis (1 RCT, 4 non-RCTs and 6 cohort studies) indicates that intrauterine infusions of G-CSF can improve endometrial thickness, CPR and ER in women undergoing IVF with thin endometrium, while it could decrease cycle cancelation rate. While, there was inconsistency, in 2017 by Li et al. [12] observed in a meta-analysis in women undergoing IVF (1 IVF with thin endometrium, 1 unselected IVF and 1 IVF with RIF) that intrauterine infusions of G-CSF was significantly correlated with a higher CPR compared with the control group(RR = 1.563, 95%CI: 1.122, 2.176). Among patients undergoing IVF with thin endometrium or RIF, ER and BPR were also significantly increased in the G-CSF group (ER: RR 1.887, 95% CI: 1.256, 2.833; BPR: RR = 2.385, 95% CI: 1.414, 4.023). However, no statistical significance was found in endometrial thickness. The inconsistency of the results with our study may be related to the complex population included in Li′s study. More RCTs in women with thin endometrium undergoing IVF were needed in measuring the effect of G-CSF on endometrium thickness and other pregnancy outcomes.

### G-CSF in women with a history of RIF undergoing IVF

Repeated implantation failure (RIF) was generally defined as failure of three fresh or frozen in vitro fertilization (IVF) cycles in which one or two high-grade quality embryos were transferred to the patient in each cycle [69]. In this meta-analysis, 14 studies included 1387 women undergoing IVF with a history of RIF, in which RIF was defined as at least three episodes of implantation failure in nine studies and as at least two episodes of implantation failure in five studies. G-CSF increased the CPR, the ER and the BPR in patients undergoing IVF with a history of RIF. The subgroup analysis suggested that CPR increased in the G-CSF group in both FET cycles (RR 1.58; 95% CI, 1.18, 2.11; supplemental Fig. 4) and ET cycles (RR 1.98; 95% CI, 1.55, 2.54, supplemental Fig. 4), thus revealing that CPR was higher in ET cycles. Besides, both subcutaneous injection (RR 2.13; 95% CI, 1.68,2.69; supplemental Fig. 3) and intrauterine injection (RR 1.71; 95% CI, 1.35,2.16; supplemental Fig. 3) of G-CSF could be beneficial for CPR, and subcutaneous injection was indicated as the more beneficial administration. Likewise, numerous studies claimed a beneficial role of G-CSF on pregnancy outcome in women undergoing IVF with a history of RIF [9, 10, 14, 17, 22, 27].

### Other outcomes in women with fertility problem

One retrospective case–control study about recurrent spontaneous abortion(RSA) women undergoing IVF and two RCT studies about RSA women after natural conception analyzing the effectiveness of G-CSF were excluded in this study. Scarpellini F et al. [61] explored the use of G-CSF for the treatment of unexplained RSA after natrual conception in one RCT. In the G-CSF group, 29 out of 35 (82.8%) women delivered a healthy baby, whereas this figure was only 16 out of 33 (48.5%) (*P* = 0.0061, OR 5.1; 95% CI 1.5, 18.4) in the control group. Santjohanser et al. [62] suggested that pregnancy rate and live birth rate increased in the G-CSF group compared with the control group in RSA patients undergoing IVF in one retrospective cohort study. Besides, Zafardoust S et al. [63] drew an inconsistent conclusion in another RCT, without any significant differences between the G-CSF group and the control group in terms of pregnancy outcomes. More RCTs should be further performed to clear the therapeutic effect of G-CSF in women with RSA.

For twin pregnancy, ectopic pregnancy, pregnancy complication, premature birth and side effects rate, there is only respectively one study involved in one outcome, so a meta-analysis is difficult to conduct. Whether the use of G-CSF can have a positive effect on the above outcomes remains unclear.

### Limitations of this study

Although large sample sizes were included in this meta-analysis, some limitations are clear. First, no RCTs were included in the meta-analysis for women undergoing IVF with RSA. Since the lack of data, a more thorough exploration of the possible effects G-CSF on pregnancy outcomes was limited. Second, in the above included RCTs, heterogeneity was observed (e.g., different administration and dosage of use of G-CSF, different embryo transfer cycle and inconsistent standard definition of pregnancy outcome). Besides, there were rare statistics about oocyte retrieval, good quality embryo, adverse events and complications of G-CSF in the existing studies, so a meta-analysis could not be conducted on the safety of G-CSF.

## Conclusion

G-CSF can improve CPR and endometrial thickness in patients with thin endometrium undergoing IVF. G-CSF can improve BPR, ER and CPR in women with a history of RIF undergoing IVF. G-CSF can improve CPR whether in FET cycle or ET cycle by subcutaneous injection or intrauterine injection for women with a history of RIF undergoing IVF. For general IVF patients, no benefit was seen with the use of G-CSF.

### Attestation statements

We claim data regarding any of the subjects in the study has not been previously published unless specified. And data is available to the editors of the journal for review or query upon request.

## Supplementary Information

**Additional files 1:** **Supplemental Figure 1.** Risk of bias graph: review authors’ judgements about each risk of bias item presented as percentages across all included studies. Supplemental Figure2 Risk of bias summary: review authors’ judgements about each risk of bias item for each included study. Supplemental Figure3 Forest plot of comparison: G-CSF vs control in RIF women for different routes of administration, outcome: Clinical pregnancy rate. Supplemental Figure4 Forest plot of comparison: G-CSF vs control in RIF women for different embryo transfer cycle, outcome: Clinical pregnancy rate. Supplemental Figure5 Forest plot of comparison: G-CSF vs control, outcome: Ongoing pregnancy rate. Supplemental Figure6 Forest plot of comparison: G-CSF vs control, outcome: Biochemical pregnancy rate.,Supplemental Figure7 Forest plot of comparison: G-CSF vs control, outcome: Embryo implantation rate. Supplemental Figure8 Forest plot of comparison: G-CSF vs control, outcome: Endometrium thickness. Supplemental Figure9 Funnel plot of comparison: G-CSF vs contro. Supplemental table 1 characteristics of included studies. Supplemental table 2 Comparison: G-CSF versus control in reproductive women with fertility problem. Supplemental table3 Characteristics of excluded studies

## Data Availability

Data can be obtained from authors on reasonable request.

## Abbreviations

G-CSF: Granulocyte colony-stimulating factor
RCTs: Randomized control trials
ART: Assisted reproduction techniques
NK: Natural killer
IVF: In vitro fertilization
ICSI: Intracytoplasmic sperm injection
RIF: Repeated implantation failure
RSA: Recurrent spontaneous abortion
RPL: Recurrent pregnancy loss
RR: Risk ratio
OR: Odds ratio
CIs: Confidence intervals
MD: Mean difference
