# Supplementary material for: Efficacy of granulocyte colony-stimulating factor for infertility undergoing IVF: a systematic review and meta-analysis
Source: Reprod Biol Endocrinol. 2023 Apr 3;21:34. doi: 10.1186/s12958-023-01063-z (PMC10069139; doi:10.1186/s12958-023-01063-z)
Supplement: Supplementary file 1 — Additional files 1: Supplemental Figure 1. Risk of bias graph: review authors’ judgements about each risk of bias item presented as percentages across all included studies. Supplemental Figure2 Risk of bias summary: review authors’ judgements about each risk of bias item for each included study. Supplemental Figure3 Forest plot of comparison: G-CSF vs control in RIF women for different routes of administration, outcome: Clinical pregnancy rate. Supplemental Figure4 Forest plot of comparison: G-CSF vs control in RIF women for different embryo transfer cycle, outcome: Clinical pregnancy rate. Supplemental Figure5 Forest plot of comparison: G-CSF vs control, outcome: Ongoing pregnancy rate. Supplemental Figure6 Forest plot of comparison: G-CSF vs control, outcome: Biochemical pregnancy rate.,Supplemental Figure7 Forest plot of comparison: G-CSF vs control, outcome: Embryo implantation rate. Supplemental Figure8 Forest plot of comparison: G-CSF vs control, outcome: Endometrium thickness. Supplemental Figure9 Funnel plot of comparison: G-CSF vs contro. Supplemental table 1 characteristics of included studies. Supplemental table 2 Comparison: G-CSF versus control in reproductive women with fertility problem. Supplemental table3 Characteristics of excluded studies [file 12958_2023_1063_MOESM1_ESM.docx]

**Additional files**


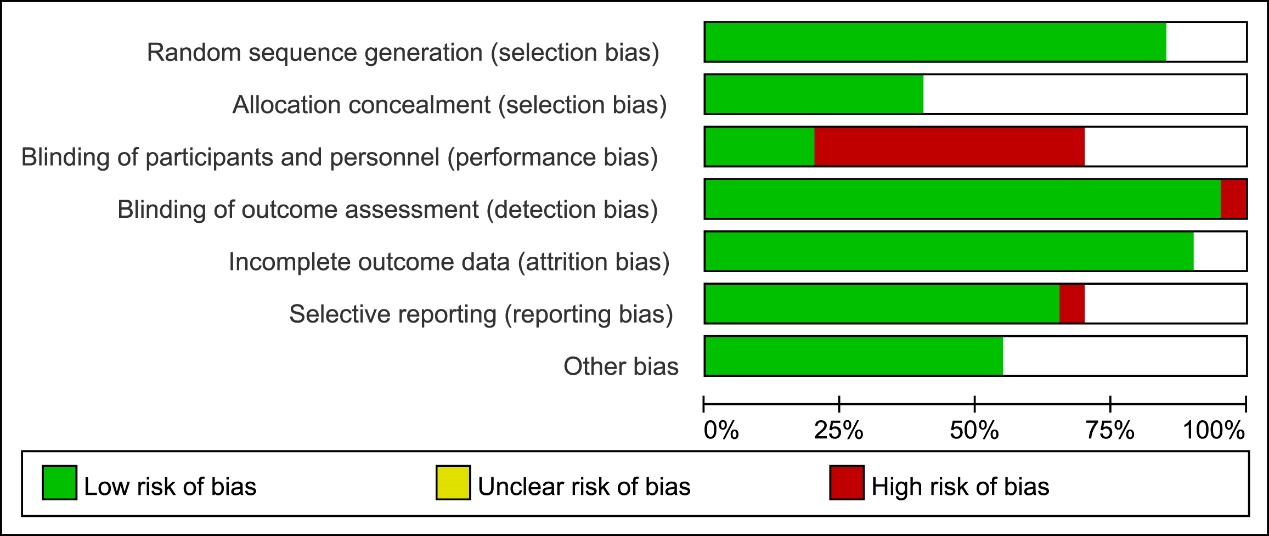


Supplemental Figure1 Risk of bias graph: review authors' judgements about each risk of bias item presented as percentages across all included studies.


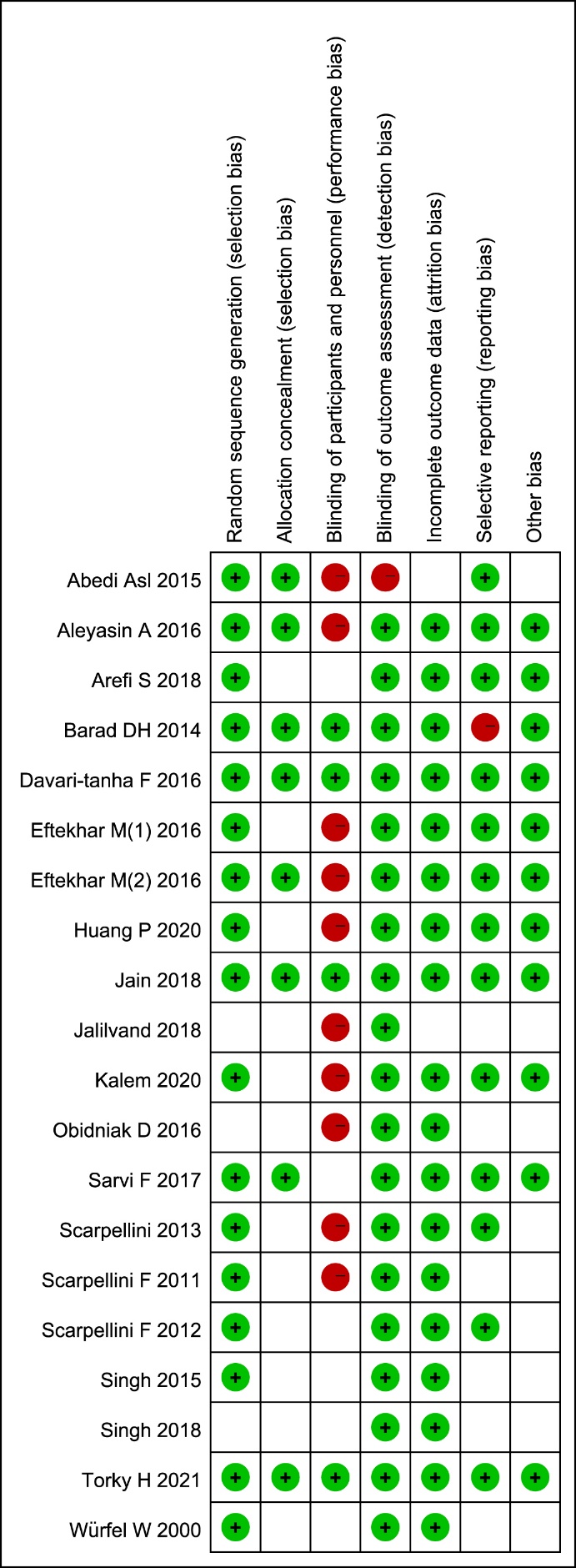


Supplemental Figure2 Risk of bias summary: review authors’ judgements about each risk of bias item for each included study.


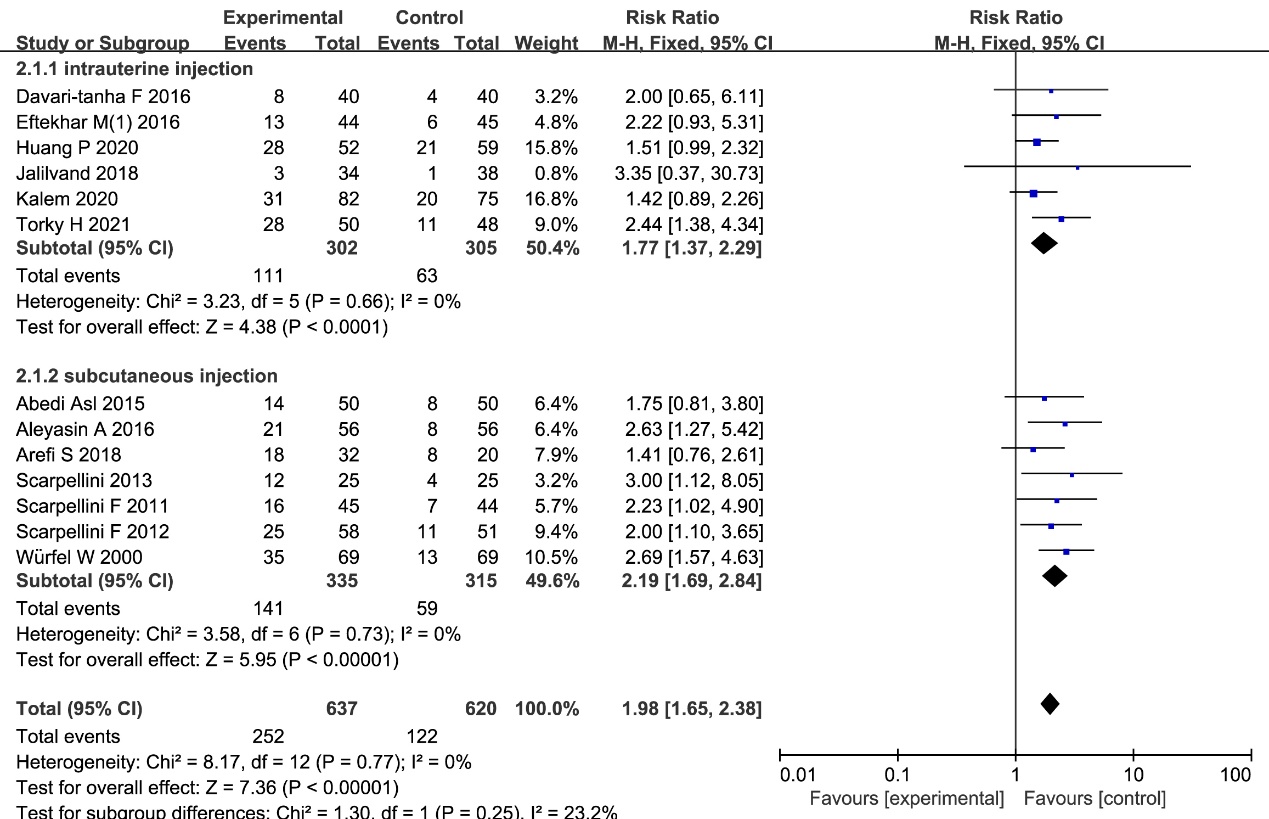


Supplemental Figure3 Forest plot of comparison: G-CSF vs control in RIF women for different routes of administration, outcome: Clinical pregnancy rate


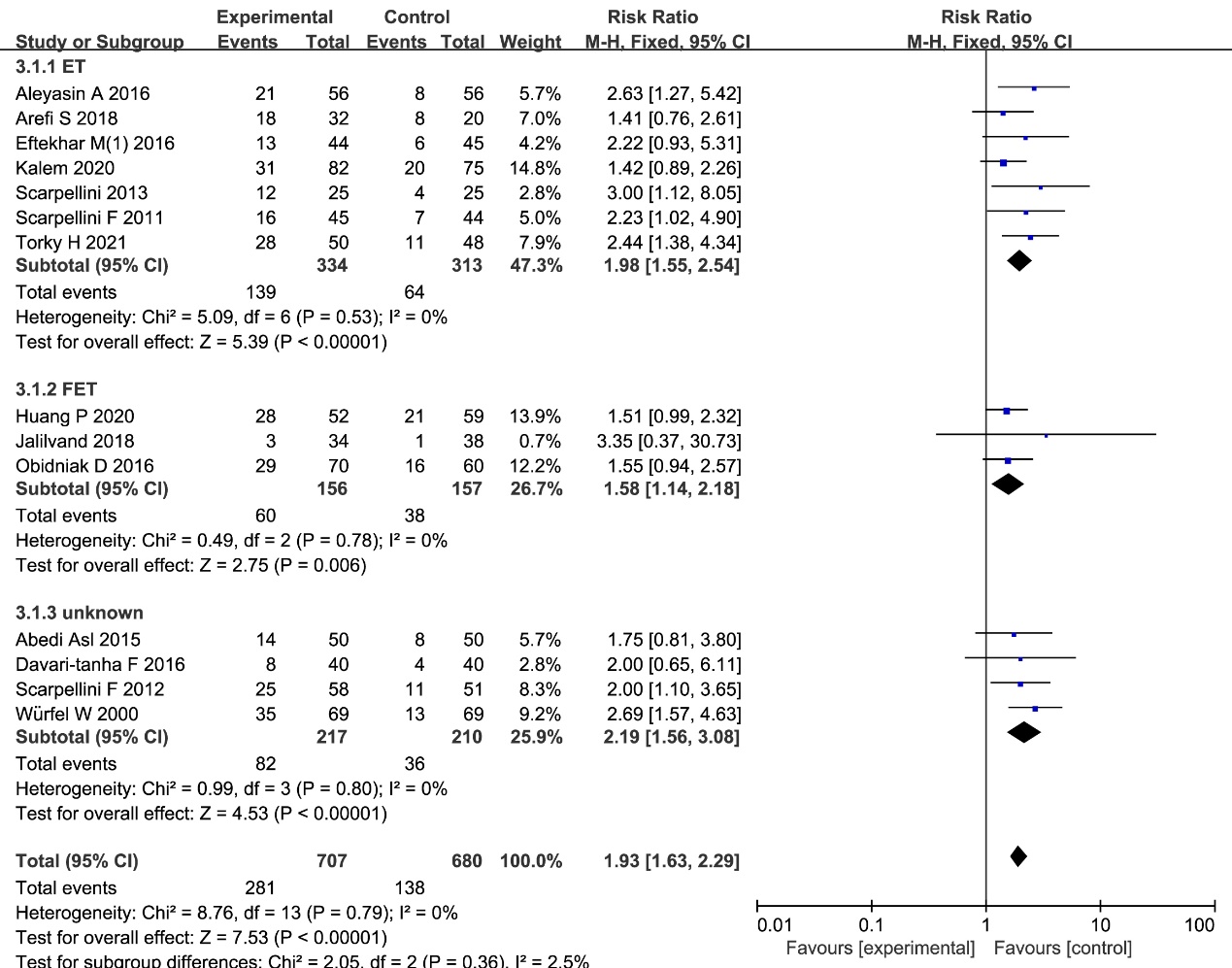


Supplemental Figure4 Forest plot of comparison: G-CSF vs control in RIF women for different embryo transfer cycle, outcome: Clinical pregnancy rate


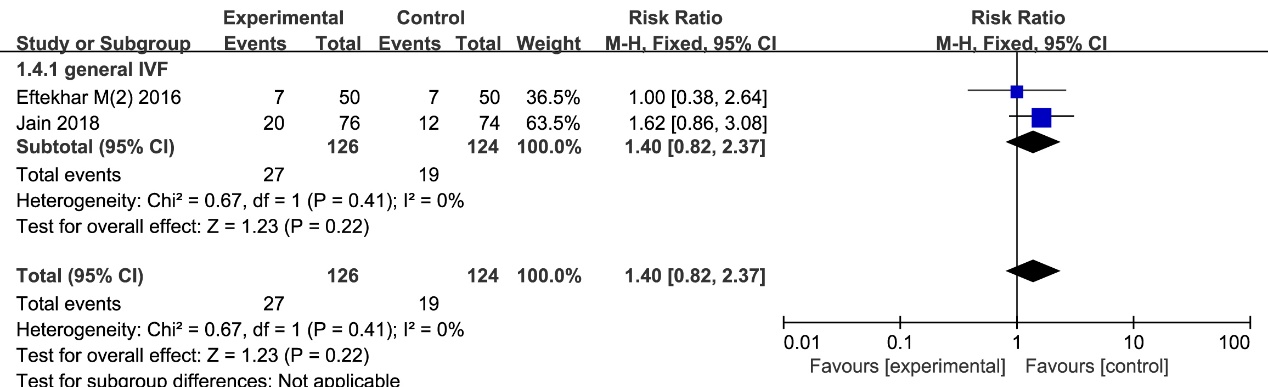


Supplemental Figure5 Forest plot of comparison: G-CSF vs control, outcome: Ongoing pregnancy rate


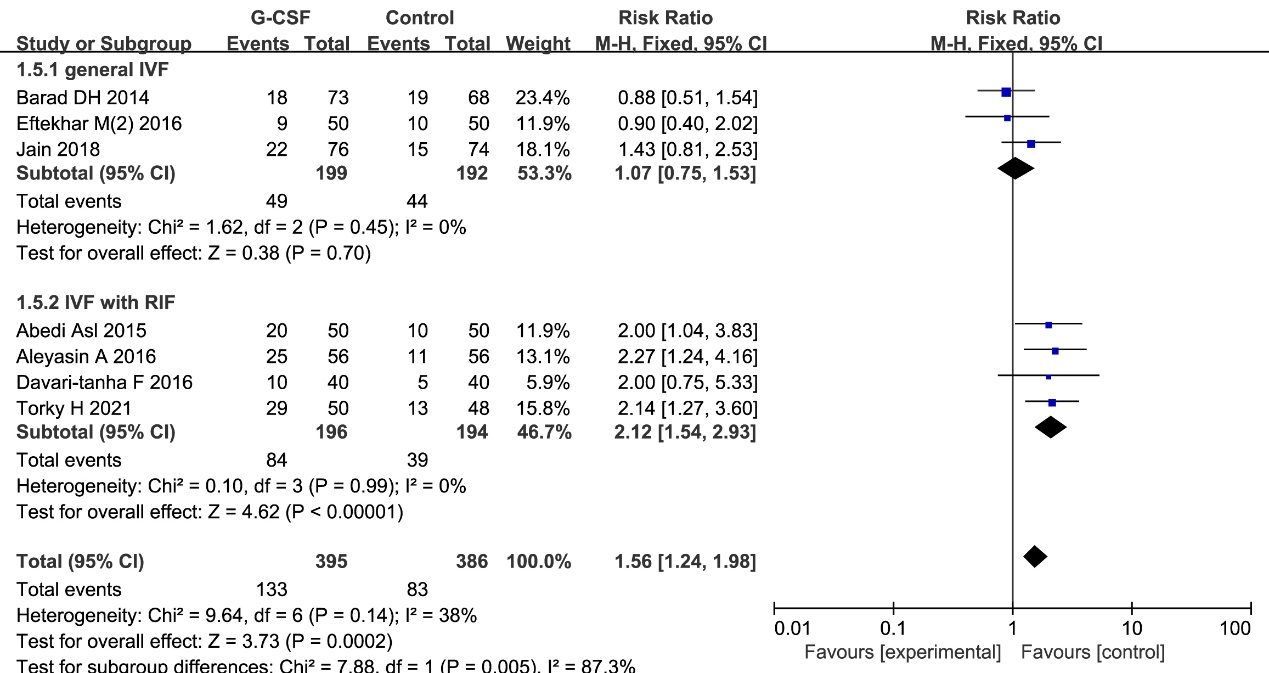


Supplemental Figure6 Forest plot of comparison: G-CSF vs control, outcome: Biochemical pregnancy rate


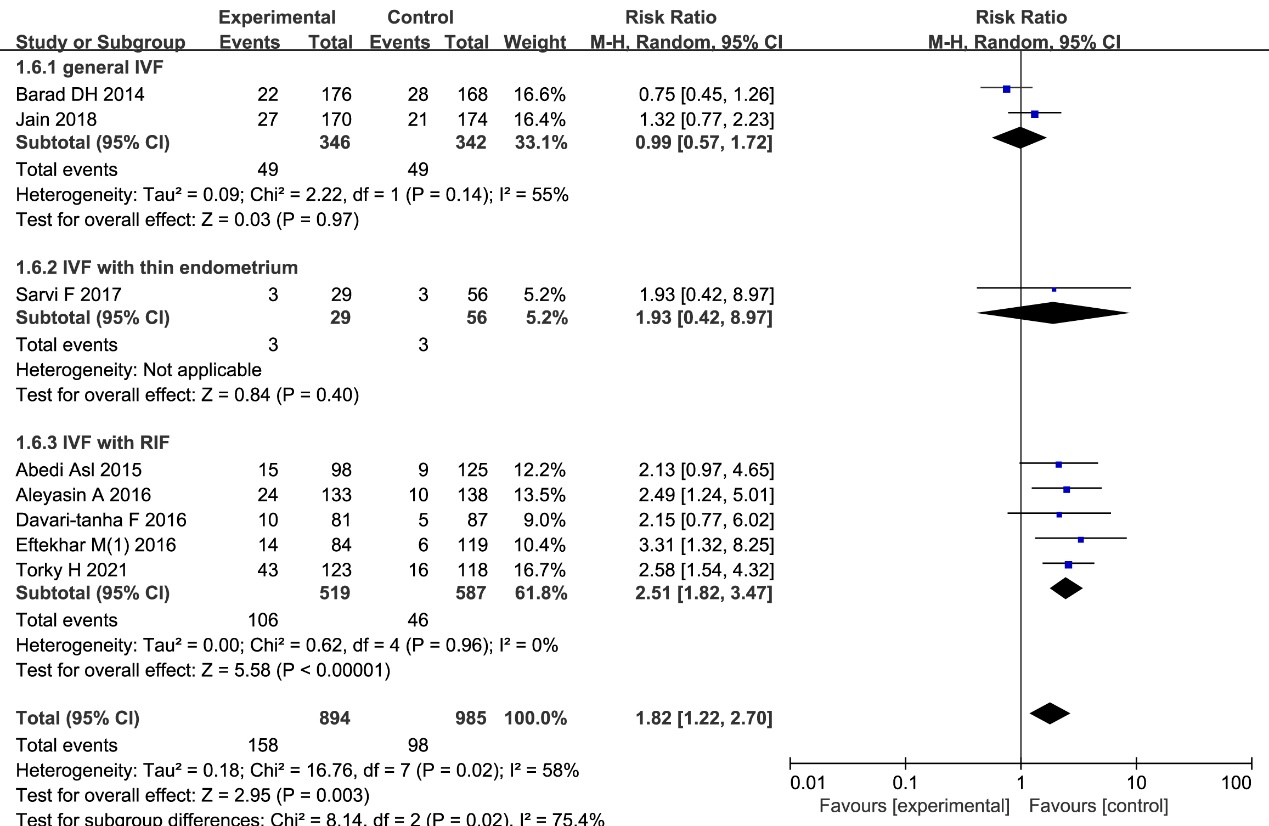


Supplemental Figure7 Forest plot of comparison: G-CSF vs control, outcome: Embryo implantation rate


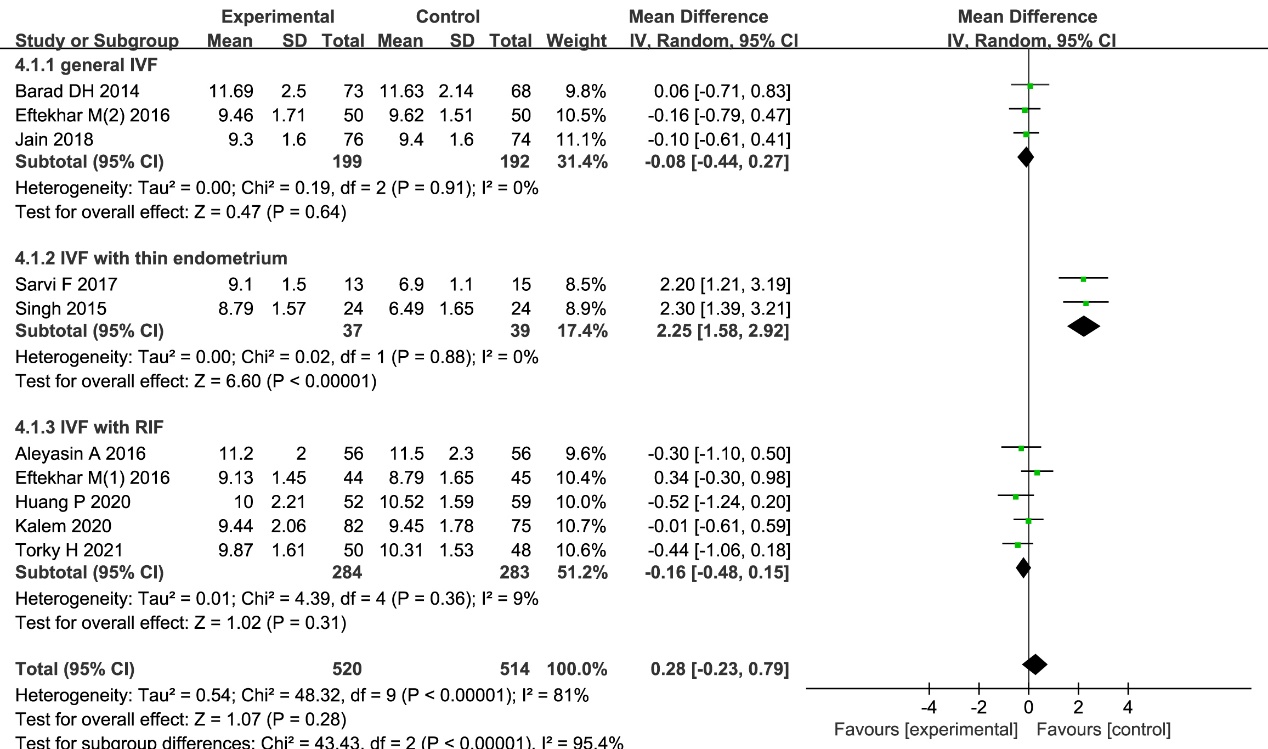


Supplemental Figure8 Forest plot of comparison: G-CSF vs control, outcome: Endometrium thickness


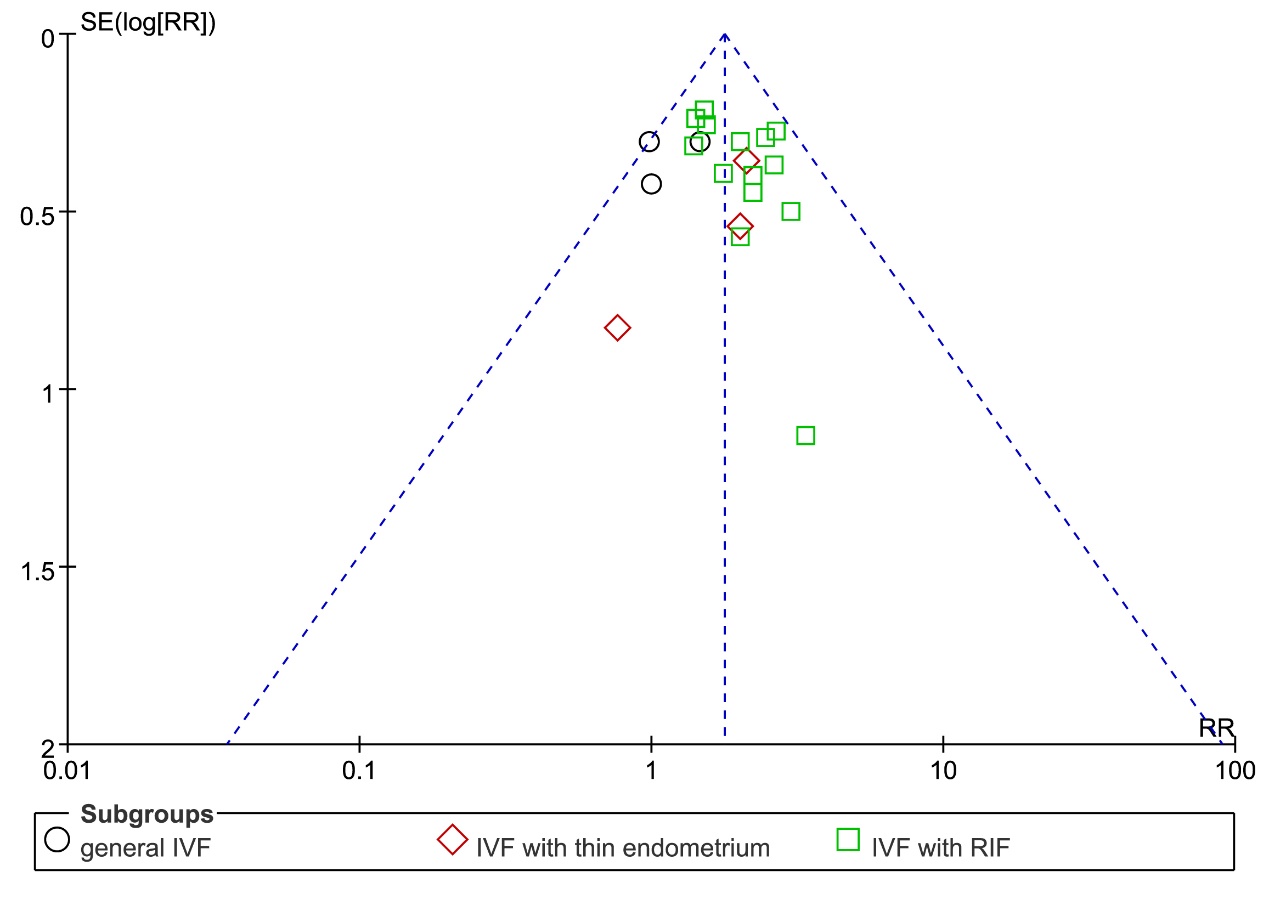


Supplemental Figure9 Funnel plot of comparison: G-CSF vs control

Supplemental table 1 characteristics of included studies

| **Study** | **Country** | **Patients** | **Diagnosis** | **Age** | **Sample size（G/C)** | **Intervention** | **Control** | **FET/**  **ET** | **Outcome** |
| --- | --- | --- | --- | --- | --- | --- | --- | --- | --- |
| Sarvi F 2017 | Iran | thin endometrium | thin endometrium unresponsive to treatment | 18 -40 | 13/15 | 300 μg/ ml G-CSF, intrauterine, on the day of hCG trigger. If endometrial thickness was < 6 mm, a second dosage of G-CSF was injected 2 to 3 days after oocyte retrieval day | 1ml saline, intrauterine | ET | CPR, ER, twin pregnancy rate |
| Singh 2015 | India | thin endometrium | thin endometrium unresponsive to treatment | - | 24/24 | 300 μg/mL G-CSF, intrauterine, on day of trigger, repeated after 48 hours if endometrial thickness was < 7 mm | Saline, intrauterine, on day of trigger | ET | CPR |
| Singh 2018 | India | thin endometrium | - | - | 56/56 | 300 μg/mL G-CSF, intrauterine or subcutaneous, on day of trigger, repeated after 48 hours if endometrial thickness was < 7 mm | Saline, intrauterine or subcutaneous | - | CPR |
| Aleyasin A 2016 | Iran | RIF | failure of implantation in at least 3 consecutive IVF attempts in which 3 high-grade embryos were transferred in each cycle | < 40 | 56/56 | 300 µg G-CSF, subcutaneous, 1 hour before embryo transfer | no treatment | ET | ER, BPR, CPR, ectopic pregnancy rate |
| Arefi S 2018 | Iran | RIF | more than three previous IVF/ICSI failures | 22-44 | 32/20 | 300 µg (0.5 ml) G-CSF, subcutaneous, 30 min before blastocyst embryo transfer | received routine procedure | ET | CPR,AR,LR , twin pregnancy rate |
| Davari-tanta F 2016 | Iran | RIF | 3 times implantation failure when there was history of transferring at least 4 good-quality embryos | < 40 | 40/40 | 300 µg(1ml) ,G-CSF, intrauterine, at the time of oocyte retrieval/ at the day of starting progesterone in FET cycle. | a catheter was passed through the cervix without any injection | FET/ET | ER, BPR, CPR, AR |
| Wurfel W 2000 | Germany | RIF | at least 3 previous failed IVF attempts with at least five embryos and more than 50% embryos transferred in good quality | < 40 | 69/69 | 300ug G-CSF, subcutaneous | - | - | CPR, AR |
| Scarpellini F 2012 | Italy | RIF | at least three previous failed IVF attempts where at least 7 good embryos were transferred | < 39 | 58/51 | 60 mg G-CSF, subcutaneous, from the day of transfer to the day of hCG test, and if it was positive the treatment was continued for other 40 days daily; | Saline, subcutaneous | - | CPR |
| Scarpellini F 2011 | Italy | RIF | at least 3 failed embryo transfers with 7 good embryos transferred | < 39 | 45/44 | 1.5 mg/kg/daily (60 to 100 mg) G-CSF, subcutaneous, from day of transfer till day of result and continued for another 40 days if positive | saline, subcutaneous | ET | CPR |
| Eftekhar M(1) 2016 | Iran | RIF | at least 2 implantation failures | 20-40 | 44/45 | 300 µg (0.5ml) G-CSF, intrauterine, after ovarian puncture | no treatment | ET | ER, CPR |
| Abedi Asl 2015 | Iran | RIF | normal endometrial thickness who had ≥ 2 implantation failure after IVF cycles | - | 50/50 | 300 µg G-CSF, subcutaneous, before implantation | - | - | ER, BPR, CPR |
| Scarpellini F 2013 | Italy | RIF | at least 3 failed ET with at least 8 good embryos transferred | < 39 | 25/25 | 60 μg G-CSF, subcutaneous, from day of transfer till day of results daily | saline,  subcutaneous | ET | CPR |
| Kalem 2020 | Turkey | RIF | Failure to achieve a clinical pregnancy after the transfer of at least four good quality embryos in a minimum of three transfer cycles | < 40 | 82/75 | 30 mIU/mL G-CSF , intrauterine, once a day on hCG day, before hCG injection | 1ml saline intrauterine | ET | CPR, AR, LR, premature birth rate |
| Obidniak D 2016 | Russia | RIF | at least 2 cycles of IVF in which good-quality embryos were transferred in each cycle without achieving a clinical pregnancy | 32 - 40 | 70/60 | Group 1 (N = 40): 30 mIU, 1 mL G-CSF, intrauterine, 5 days prior to embryo transfer Group 2 (N = 30): 30 mIU, 1 mL G-CSF, subcutaneous | no treatment | FET | CPR |
| Huang P 2020 | China | RIF | ≥two failed implantations (each time containing at least one high-quality embryo) BMI 18–24;  normal endometrial thickness (8–16 mm) | ≤38 | 52/59 | 150 mg, 1 ml G-CSF, intrauterine | normal saline | FET | CPR, LR, AR |
| Torky H 2021 | Egypt | RIF | recurrent implantation failure (three or more failed attempts with at least four good quality embryos transferred) | 20 -39 | 50/48 | 100 µg G-CSF, intrauterine, after oocyte retrieval | saline | ET | ER, BPR, CPR, AR |
| Jalilvand 2018 | Iran | RIF | with a history of 2 or more implantation failures (RIF) | 18 - 45 | 34/38 | 100μg G-CSF, intrauterine, on the day of progesterone until ET | no treatment | FET | CPR |
| Eftekhar M(2) 2016 | Iran | IVF | Normal endometrial thickness excluded RIF | 18 -40 | 50/50 | 300μg G-CSF, intrauterine, at the oocyte retrieval day | no treatment | ET | CPR, BPR, OPR, AR |
| Barad DH 2014 | USA | IVF | unselected IVF | 18 -38 | 73/68 | 300 μg/1.0 mL G-CSF, intrauterine, on the morning of hCG administration before noon | normal saline | FET  /ET | CPR, BPR, ER, AR |
| Jain 2018 | India | IVF/ICSI | excluded poor ovarian reserve | 21 - 38 | 76/74 | 300μg (0.5 mL) G-CSF, intrauterine, on the day of ovulation trigger | 0.5 mL saline, intrauterine | ET | CPR, BPR, OPR, ER |

-:unknown

Supplemental table 2 Comparison: G-CSF versus control in reproductive women with fertility problem

| **Outcome or Subgroup** | **Studies** | **Participants** | **Statistical Method** | **Effect Estimate** |
| --- | --- | --- | --- | --- |
| **1.1 clinical pregnancy** | 20 | 1966 | Risk Ratio (M-H, Fixed, 95% CI) | 1.77 [1.52, 2.05] |
| 1.1.1 general IVF | 3 | 391 | Risk Ratio (M-H, Fixed, 95% CI) | 1.16 [0.80, 1.69] |
| 1.1.2 IVF with thin endometrium | 3 | 188 | Risk Ratio (M-H, Fixed, 95% CI) | 1.85 [1.07, 3.18] |
| 1.1.3 IVF with RIF | 14 | 1387 | Risk Ratio (M-H, Fixed, 95% CI) | 1.93 [1.63, 2.29] |
| **1.2 live birth** | 3 | 320 | Risk Ratio (M-H, Fixed, 95% CI) | 1.51 [0.82, 2.78] |
| 1.2.1 IVF with RIF | 3 | 320 | Risk Ratio (M-H, Fixed, 95% CI) | 1.51 [0.82, 2.78] |
| **1.3 abortion** | 9 | 377 | Risk Ratio (M-H, Fixed, 95% CI) | 0.65 [0.39, 1.09] |
| 1.3.1 general IVF | 3 | 90 | Risk Ratio (M-H, Fixed, 95% CI) | 0.51 [0.19, 1.39] |
| 1.3.2 IVF with RIF | 6 | 287 | Risk Ratio (M-H, Fixed, 95% CI) | 0.71 [0.39, 1.31] |
| **1.4 ongoing pregnancy** | 2 | 250 | Risk Ratio (M-H, Fixed, 95% CI) | 1.40 [0.82, 2.37] |
| 1.4.1 general IVF | 2 | 250 | Risk Ratio (M-H, Fixed, 95% CI) | 1.40 [0.82, 2.37] |
| **1.5 biochemical pregnancy** | 7 | 781 | Risk Ratio (M-H, Fixed, 95% CI) | 1.56 [1.24, 1.98] |
| 1.5.1 general IVF | 3 | 391 | Risk Ratio (M-H, Fixed, 95% CI) | 1.07 [0.75, 1.53] |
| 1.5.2 IVF with RIF | 4 | 390 | Risk Ratio (M-H, Fixed, 95% CI) | 2.12 [1.54, 2.93] |
| **1.6 embryo implantation** | 8 | 1879 | Risk Ratio (M-H, Random, 95% CI) | 1.82 [1.22, 2.70] |
| 1.6.1 general IVF | 2 | 688 | Risk Ratio (M-H, Random, 95% CI) | 0.99 [0.57, 1.72] |
| 1.6.2 IVF with thin endometrium | 1 | 85 | Risk Ratio (M-H, Random, 95% CI) | 1.93 [0.42, 8.97] |
| 1.6.3 IVF with RIF | 5 | 1106 | Risk Ratio (M-H, Random, 95% CI) | 2.51 [1.82, 3.47] |
| **2.1 clinical pregnancy** | 15 | 1447 | Risk Ratio (M-H, Fixed, 95% CI) | 1.91 [1.62, 2.26] |
| 2.1.1 intrauterine | 7 | 707 | Risk Ratio (M-H, Fixed, 95% CI) | 1.71 [1.35, 2.16] |
| 2.1.2 subcutaneous | 8 | 740 | Risk Ratio (M-H, Fixed, 95% CI) | 2.13 [1.68, 2.69] |
| **3.1 clinical pregnancy** | 14 | 1387 | Risk Ratio (M-H, Fixed, 95% CI) | 1.93 [1.63, 2.29] |
| 3.1.1 ET | 7 | 647 | Risk Ratio (M-H, Fixed, 95% CI) | 1.98 [1.55, 2.54] |
| 3.1.2 FET | 3 | 313 | Risk Ratio (M-H, Fixed, 95% CI) | 1.58 [1.14, 2.18] |
| 3.1.3 unknown | 4 | 427 | Risk Ratio (M-H, Fixed, 95% CI) | 2.19 [1.56, 3.08] |
| **4.1 endometrium thickness** | 10 | 1034 | Mean Difference (IV, Random, 95% CI) | 0.28 [-0.23, 0.79] |
| 4.1.1 general IVF | 3 | 391 | Mean Difference (IV, Random, 95% CI) | -0.08 [-0.44, 0.27] |
| 4.1.2 IVF with thin endometrium | 2 | 76 | Mean Difference (IV, Random, 95% CI) | 2.25 [1.58, 2.92] |
| 4.1.3 IVF with RIF | 5 | 567 | Mean Difference (IV, Random, 95% CI) | -0.16 [-0.48, 0.15] |

Supplemental table3 Characteristics of excluded studies

| **study** | **participants** | **Reason for exclusion** |
| --- | --- | --- |
| Zhu YC 2021 | thin endometrium | Retrospective cohort Study |
| Xu B 2015 | thin endometrium | case-control |
| Li Y 2014 | thin endometrium | case-control |
| Eftekhar M 2014 | thin endometrium | case-control |
| Lian RC 2019 | thin endometrium | retrospective cohort analysis |
| [Gleicher N 2013](https://pubmed.ncbi.nlm.nih.gov/?term=Gleicher+N&cauthor_id=23081869) | thin endometrium | cohort study |
| Szlarb 2014 | thin endometrium | retrospective cohort study |
| Kunicki M 2016 | thin endometrium | case-control |
| Santjohanser 2013 | RSA | Retrospective cohort study |
| Scarpellini F（1）2009 | RSA | Randomized controlled trial (RCT)，natural conception |
| Zafardoust S 2017 | RSA | Randomized controlled trial (RCT)，natural conception |
| Zeyneloglu H 2019 | RIF | retrospective case–control study |
| Scarpellini F 2009 | IVF | Randomized controlled trial (RCT)，low ovarian responder, and FSH ≥ 15 UI/L |
